# How to Reform Our Reformatories

**Published:** 1903-04-04

**Authors:** 


### HOW TO REFORM OUR REFORMATORIES.

THE reform advocated by the Rev. W. Douglas Morrison (late chaplain of Wandsworth Prison) included a step forward in the management of these institutions, and the first and most vital step was the improvement in the quality of their officials. These institutions required men of higher education and of sterling character. The only way in which to effect this improvement was by removing the whole of these establishments from the penal department and placing them under the control of the Education Department. Children under 16 years of age could not be considered criminals, and therefore should not have criminal or penal treatment. Dr. Morrison quoted in support of his contention the report of Sir Godfrey Lushington's committee, in which it was stated that the Education Department was willing to accept the management of these institutions, and that in the opinion of the committee this department was better qualified to deal with these establishments than the Home Office.

